# No Association Observed Between the Number of Infectious Disease Experts and Prevalence of Antimicrobial-Resistant Pathogens in Japan

**DOI:** 10.7759/cureus.16918

**Published:** 2021-08-05

**Authors:** Hideharu Hagiya, Fumio Otsuka

**Affiliations:** 1 Department of General Medicine, Okayama University Graduate School of Medicine, Dentistry and Pharmaceutical Sciences, Okayama, JPN

**Keywords:** antimicrobial resistance, certified nurses in infection control, infection prevention and control, infectious disease, japan nosocomial infections surveillance

## Abstract

Introduction: The global spread of emerging infections has increased the demand for infectious disease (ID) experts. There is no established method to evaluate the sufficiency of professionals on a regional basis. We aimed to determine the correlation of the number of ID doctors and certified nurses in infection control (CNIC) with the prevalence of representative antimicrobial-resistant (AMR) pathogens across the 47 prefectures in Japan using publicly available databases.

Methods: We determined the number of ID doctors and CNIC registered in each prefecture based on the Japanese Association for Infectious Diseases and the Japanese Nursing Association websites and calculated their numbers per 100,000 population. Data on representative AMR pathogens were extracted from the Japan Nosocomial Infections Surveillance database. Spearman’s correlation coefficient was used to measure statistical associations.

Results: There was no epidemiologically applicable correlation between the deployment of ID doctors and CNIC and the isolation rates of methicillin-resistant *Staphylococcus aureus*, vancomycin-resistant *Enterococcus faecium,* cefotaxime- or levofloxacin-resistant *Escherichia coli* and *Klebsiella pneumoinae, *and meropenem-resistant *Pseudomonas aeruginosa*. Solely, the isolation rate of levofloxacin-resistant* K. pneumoinae *and the number of CNIC were statistically correlated (correlation coefficient = −0.33; *p *= 0.02), while the isolation rate of cefotaxime-resistant *E. coli* was paradoxically correlated with the number of ID doctors (correlation coefficient = 0.33; *p *= 0.02)*.*

Conclusions: Our macroscopic analysis using the open database was not a reliable method to evaluate the sufficiency of ID experts across the prefectures in Japan. A scheme to assess the appropriate distribution of ID experts should be developed.

## Introduction

With the emergence of a global pandemic of antimicrobial resistance (AMR) [1], a well-organized platform for infection prevention and control (IPC) activities should be established. Infectious disease (ID) doctors and certified nurses in infection control (CNIC) play a crucial role in medical IPC activities. However, these populations are limited in Japan. As of April 1, 2021, there were 1,622 board-certified physicians of the Japanese Association for Infectious Diseases [2]. The Japanese Nursing Association has accredited 2,977 CNIC as of December 2020 [3]. The Japanese Association for Infectious Diseases stated that each medical institute with over 300 beds should have at least one full-time ID doctor on duty and required 3,000 to 4,000 ID doctors working in Japanese hospitals [4]. The Centers for Disease Control and Prevention of the United States stated that medical institutes should employ one CNIC per 250 hospital beds [5]. Medical staff education and training need to be improved to produce more ID experts to achieve these recommendations.

While IPC activities are narrowly defined on a hospital-based approach, its approach should be broader and region-based because the AMR pathogens are transported among medical institutes through patient transfers. An increase in the number of ID experts is expected to decrease the AMR pathogens in a region. However, to the best of our knowledge, there is no consensus on the acceptable methods to verify or confirm this assumption.

The Japan Nosocomial Infections Surveillance (JANIS) data have been made publicly available by the Japanese Ministry of Health, Labor, and Welfare; JANIS was launched in 2000 as a sustainable surveillance model for AMR prevalence. It is one of the most extensive national AMR surveillance worldwide [6,7]. It can be used as a tool for inter-prefectural surveillance of AMR pathogens [8] and automated AMR detection systems [9]. We investigated the role of macroscopic data as an indicator of the sufficiency of ID experts in each prefecture in Japan. This study aimed to determine the correlation of the number of ID doctors and CNIC with the isolation rates of representative AMR pathogens across 47 prefectures in Japan.

## Materials and methods

This descriptive study used publicly available data. Ethics committee approval and the need for informed consent were waived by the institutional review board of the Okayama University Hospital because the detailed data on individuals and hospitals were all anonymized (No. 1910-009). An ID doctor was defined as a board-certified physician of the Japanese Association for Infectious Diseases. We collected data on the number of ID doctors in each prefecture (as of April 1, 2021) from the website of the Japanese Association for Infectious Diseases [2]. CNICs were defined as those accredited by the Japanese Nursing Association, and we collected data on the number of CNICs as of December 2020 from their official website [3]. We gathered population data from the website of the Statistics Bureau of the Ministry of Internal Affairs and Communications as of 2019. Using these figures, we calculated the number of ID doctors and CNIC per 100,000 population in each prefecture.

We then compared the prefecture-wide isolation rates of AMR pathogens as a comparative parameter. The rates of methicillin-resistant *Staphylococcus aureus* (MRSA), vancomycin-resistant *Enterococcus faecium* (VRE), third-generation cephalosporin (cefotaxime; CTX)-resistant *Escherichia coli*, fluoroquinolone (levofloxacin; LVFX)-resistant *E. coli*, CTX-resistant *Klebsiella pneumoniae*, LVFX-resistant *K. pneumoniae*, and carbapenem (meropenem; MEPM)-resistant *Pseudomonas aeruginosa* were collected from the JANIS database [10]. MRSA was defined as an *S. aureus* strain resistant to either oxacillin or cefoxitin (CFX). However, in this study, no corresponding data were available, and we classified the CFX-resistant *S. aureus* strain as MRSA.

We used Spearman’s correlation coefficient to measure the statistical association between the two continuous variables: the prefecture-by-prefecture numbers of ID doctors and CNIC per 100,000 population and the isolation rates of the AMR pathogens. Statistical differences were set at a p-value <0.05.

## Results

**Figure 1:**
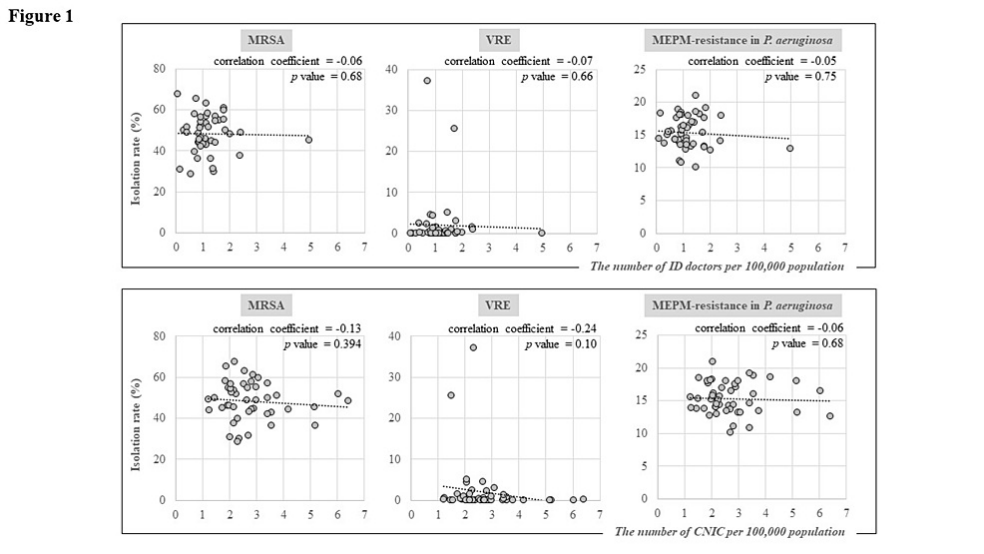
Correlation between the numbers of infectious disease specialists and certified infection control nurses in 47 prefectures in Japan and isolation rates of methicillin-resistant S. aureus, vancomycin-resistant E. faecium, and meropenem-resistant P. aeruginosa.

**Figure 2:**
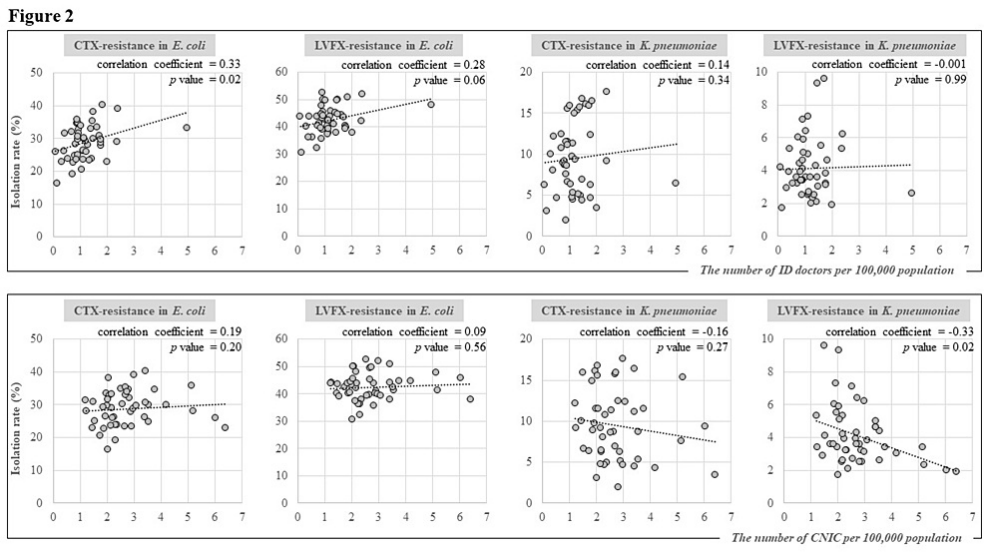
Correlation between the numbers of infectious disease specialists and certified infection control nurses in 47 prefectures in Japan and isolation rates of cefotaxime-resistant and levofloxacin-resistant E. coli and cefotaxime-resistant and levofloxacin-resistant K. pneumoniae.

As a result, there was no correlation of the deployment of ID doctors and CNIC with the isolation of MRSA, VRE, and MEPM-resistant *P. aeruginosa* (Figure 1). In addition, no correlation was observed for Enterobacteriaceae, except for CTX-resistant *E. coli* and LVFX-resistant *K. pneumoniae* (Figure 2). The isolation rates of CTX-resistant *E. coli* correlated with the number of ID doctors (correlation coefficient = 0.33; p = 0.02). The isolation rates of LVFX-resistant *K. pneumoniae* correlated with the number of CNIC (correlation coefficient = −0.33; p = 0.02).

## Discussion

In the present study, there was mostly no epidemiologically applicable correlation between the number of ID experts per population and the isolation rates of AMR pathogens. A correlation between CTX-resistant *E. coli* and ID doctors was found. However, the relationship was paradoxical; an increase in ID doctors was associated with higher pathogen isolation, suggesting a presence of confounding factors. The correlation between LVFX-resistant *K. pneumoniae* and CNIC indicated that the presence of ID experts could be associated with a reduction in AMR pathogens; however, this could be just a matter of multiple testing. In summary, our macroscopic analysis appears not ideal for estimating the sufficiency of ID experts across prefectures.

There are possible explanations for the difficulties associated with our method; these could be limitations for this research. The AMR pathogens analyzed in our study were all representative nosocomial organisms necessitating strict control via IPC activities. However, MRSA does not exclusively cause nosocomial transmission owing to the emergence of community-acquired strains [11]. In addition, extended-spectrum beta-lactamase-producing (substituted with CTX-resistant *E. coli* in this report) and fluoroquinolone-resistant *E. coli* were isolated more frequently in the outpatient setting [12]. Thus, its increasing or decreasing trends could not be directly related to the IPC activities in the hospitals. Data on carbapenem-resistant *P. aeruginosa* might be inappropriate because the detection rate was too low for the statistical analysis. Finally, not only the isolation rate but the prevalence or incidence number of each pathogen could be possible parameters.

In Japanese hospitals, the ID doctors and CNICs are necessarily not involved in full-time IPC activities; some of them work as part-time ID experts or even as one of the hospital staff in a ward, although being certified. Thus, just not the number of ID experts, but their effort rates in IPC activities should be evaluated to figure out their contribution. Additionally, as for ID doctors, their experience and knowledge on IPC activities vary because it is not a core requirement for certification. Thus, it could be invalid to generalize the involvement of all ID doctors in IPC activities. Lastly, no matter how hard they are engaged, it should certainly take time to reduce the AMR pathogens; infection control is not an overnight solution. After all, the presence of ID experts merely may not guarantee a reduction of AMR pathogens.

Countermeasures against AMR require continuous efforts from multidisciplinary perspectives, including medical, educational, epidemiological, governmental, and economic. The following issues should be primarily addressed: (i) founding a platform that enables pharmacists and clinical laboratory technicians to acquire ID certification and actively join IPC activities, (ii) obligatory full-time employment of ID doctors and CNICs at middle- to large-scale hospitals, (iii) improvement of regional imbalance of the ID experts, (iv) amendment of the educational curriculum in medical school, and (v) mandatory training for infectious diseases during postgraduate training.

## Conclusions

In summary, our macroscopic analysis, integrating certified ID experts and the JANIS database, to compare the inter-prefectural sufficiency of health professionals may be an ineffective approach. ID experts are essential to combat AMR, and continuous efforts for sustainable education are required. A multifaceted and reliable approach to evaluate the carrier development of ID experts in a particular region will be a cornerstone for establishing IPC activities to address the progressive expansion of AMR pathogens. A longitudinal study that can prove causality between an increase in ID experts and a decrease in AMR pathogens is warranted for future approaches.
